# The impact of rising sea temperatures on an Arctic top predator, the narwhal

**DOI:** 10.1038/s41598-020-75658-6

**Published:** 2020-10-29

**Authors:** P. Chambault, O. M. Tervo, E. Garde, R. G. Hansen, S. B. Blackwell, T. M. Williams, R. Dietz, C. M. Albertsen, K. L. Laidre, N. H. Nielsen, P. Richard, M. H. S. Sinding, H. C. Schmidt, M. P. Heide-Jørgensen

**Affiliations:** 1 Greenland Institute of Natural Resources, Strandgade 91, 1401 Copenhagen, Denmark; 2 Greeneridge Sciences, Inc, 5266 Hollister Avenue, Suite 107, Santa Barbara, CA 93111 USA; 3 University of California, Santa Cruz, USA; 4 Department of Bioscience, Aarhus University, Frederiksborgvej 399, 4000 Roskilde, Denmark; 5 DTU Aqua, Technical University of Denmark, 2800 Kgs. Lyngby, DK Denmark; 6 Applied Physics Laboratory, Polar Science Center, University of Washington, Seattle, WA 98105-6698 USA; 7 Greenland Institute of Natural Resources, Box 570, 3900 Nuuk, Greenland; 8 Fisheries and Oceans Canada, Winnipeg, MB R3T 2N6 Canada; 9 Smurfit Institute of Genetics, Trinity College Dublin, Dublin 2, Ireland

**Keywords:** Behavioural ecology, Population dynamics

## Abstract

Arctic top predators are expected to be impacted by increasing temperatures associated with climate change, but the relationship between increasing sea temperatures and population dynamics of Arctic cetaceans remains largely unexplored. Narwhals (*Monodon monoceros*) are considered to be among the most sensitive of Arctic endemic marine mammals to climate change due to their limited prey selection, strict migratory patterns and high site fidelity. In the context of climate change, we assume that the population dynamics of narwhals are partly influenced by changes in environmental conditions, with warm areas of increasing sea temperatures having lower abundance of narwhals. Using a unique large dataset of 144 satellite tracked narwhals, sea surface temperature (SST) data spanning 25 years (1993–2018) and narwhal abundance estimates from 17 localities, we (1) assessed the thermal exposure of this species, (2) investigated the SST trends at the summer foraging grounds, and (3) assessed the relationship between SST and abundance of narwhals. We showed a sharp SST increase in Northwest, Mideast and Southeast Greenland, whereas no change could be detected in the Canadian Arctic Archipelago (CAA) and in the Greenland Sea. The rising sea temperatures were correlated with the smallest narwhal abundance observed in the Mideast and Southeast Greenland (< 2000 individuals), where the mean summer sea temperatures were the highest (6.3 °C) compared to the cold waters of the CAA (0.7 °C) that were associated with the largest narwhal populations (> 40,000 individuals). These results support the hypothesis that warming ocean waters will restrict the habitat range of the narwhal, further suggesting that narwhals from Mideast and Southeast Greenland may be under pressure to abandon their traditional habitats due to ocean warming, and consequently either migrate further North or locally go extinct.

## Introduction

Climate change is affecting almost all regions and ecosystems, with the Arctic demonstrating the greatest, irreversible consequences on marine life. Rapid sea-ice loss and increasing temperatures^1,2^ are altering the distribution and abundance of low trophic-level organisms, generating cascading effects through the entire food chain from phytoplankton to mammalian predators^3^. Arctic marine mammals have the potential to move over long distances to adapt to changing and erratic resource availability. However, their capacity to adjust long-term adaptations, like site-fidelity and fixed migratory patterns, to climate-induced perturbations remains poorly known^4,5^.

Among Arctic cetaceans, the narwhal (*Monodon monoceros*) has a large geographic range extending from the Canadian Arctic Archipelago (CAA) to East Greenland, Svalbard and the western part of the Russian Arctic^6–11^. Narwhals are known to exhibit a high degree of site-fidelity and to be closely associated with specific migratory corridors during spring and fall movements between summer and winter grounds^12^. During winter, narwhals are mainly found in offshore areas over deep water and often in areas completely covered with pack-ice with only leads available for breathing. In spring the narwhals move towards coastal areas. The inshore summer foraging grounds, where they congregate during the open water season, are considered the primary basis for identifying separate population units of narwhals^13^.

Traditionally narwhals have been considered tightly associated with sea ice^14^, and while this may be the case for some populations most narwhal populations spend 2–3 summer and fall months in ice-free areas but always in areas dominated by cold polar waters. Narwhals are generally considered to be among the most sensitive and vulnerable of Arctic endemic marine mammals to climate changes^15^ due to their choice of habitat, limited prey selection, strict migratory patterns and high site fidelity. At the same time, narwhals are hunted in Greenland and Canada where populations are monitored closely to detect changes that may by caused by the hunting pressure or the ongoing changes in the habitats. It is therefore important to understand the current habitat selection of narwhals and the climate drivers that may potentially change their habitats.

A marked variability in abundance estimates across population units have been highlighted from aerial surveys conducted at the different summer grounds, with localities in the CAA showing a greater abundance of narwhals (> 141,000 individuals) compared to West (~ 14,400) and East Greenland (~ 6400)^16,17^. The contrasting oceanographic conditions encountered at the different summer grounds as well as the hunting pressure may explain the demographic variability for the species.

Cold and buoyant waters from the CAA enter northern Baffin Bay and are transported southward towards Davis Strait along the western part of Baffin Bay along the Canadian coast, while a warm and salty subsurface current along the West Greenland coast transports dense Atlantic waters northward towards northern Baffin Bay^18^. In comparison, East Greenland is characterized by the southward flowing East Greenland Current which transports cold water masses that are originally derived from the Arctic Ocean but are mixed with coastal runoffs from melting glaciers along East Greenland^19^. In addition, warm and saline Atlantic waters flowing northward between Iceland and East Greenland (the Irminger Current) in the Denmark Strait occasionally reach the East Greenland shelf break^20^. In a context of climate change, we therefore might assume that the population dynamics of narwhals are partly influenced by changes in environmental conditions, with warm areas with increasing sea temperatures having lower abundance of narwhals. This is supported by the narrow temperature habitat usage observed in narwhals in East Greenland^21^.

## Materials and methods

### Study areas and tag deployment

Live-capture operations of narwhals were conducted in collaboration with Inuit hunters in Northeastern Canada, West and East Greenland. Set nets of 40 to 80 m length and 5 to 8 m deep were deployed from shore to an anchor at suitable sites. Lookouts for whales were maintained from land-based promontories, from which the nets were kept under constant diurnal surveillance. As soon as there were indications of a whale being entangled, the net was released from the anchor and the whale pulled to the surface and towards the shore where instrumentation took place. Sex of the whales was determined based on presence (male) or absence (female) of a tusk. Various generations of Wildlife Computers (Redmond, Seattle, WA, USA) SLTDR, SPOT tags and FastLoc transmitters were mounted on the tusk or for the majority of the whales on the keratinized dorsal ridge of the whales with 2–3 delrin nylon pins (8 mm diameter) secured with nylon washers and bolts on each end^22,23^.

### Location processing

Location data were relayed through the Argos Data Collection and Location System and decoded using Argos Message Decoder (DAP Ver. 3.0, build 114, Wildlife Computers). The least-square filtering algorithm was used for the Argos data until 2011, after which Kalman filtering was used. All statistical analyses were performed using R software version 3.6.2^24^. The modelling approach of Albertsen et al.^25^ implemented in the R package *argosTrack*^26^ was applied to the tracking data to improve the estimation of the whale’s actual tracks from Argos locations. Tracks were estimated separately for each individual using a step-wise approach simplifying the estimation model if convergence issues were encountered, for instance for short tracks. Initially, the estimation assumed a continuous time-correlated random walk movement model with t-distributed measurement error. Variance parameters were estimated for each Argos location class. If the model did not converge properly, the ratio between variance parameters was fixed following the results of Albertsen et al.^25^. Subsequently, a multivariate normal distribution was used for the measurement error with and without free variance parameters. Finally, if a model still had not converged, a random walk movement model was used with and without free measurement variance parameters. In 2015, 2017 and 2018, Fastloc GPS transmitters were used and no estimation model was applied to their more precise GPS positions^27,28^. In order to assess temporal patterns, seasons were discriminated as follows: autumn from October to December, winter from January to March, spring from April to June and summer from July to September. To assess the influence of rising sea temperatures on narwhals we focused on the summer period which corresponds to peak of ocean temperatures in the Arctic.

### Environmental data

We extracted environmental variables from model simulations to characterize the habitat experienced by the narwhals. The associated Sea Surface Temperature (SST) and Sea Ice Concentration (SIC) data were first extracted daily from the products *Global Ocean Physics Reanalysis Glorys S2V4* (PHYS 001-024) and the *Global Ocean Physics Reanalysis Glorys12v1* (PHY-001-030) at a resolution of 0.08° (from E.U. Copernicus Marine Service Information) at each whale location. In order to generate time-series of SST and SIC between 1993 and 2018, monthly grids of both variables were also extracted from the products *Global Ocean Physics Reanalysis Glorys12v1* (PHY-001-030) and *Global Ocean and Physics Analysis Forecast* (PHY-001-024) and averaged over the extension of each of 17 summer foraging grounds with narwhal aggregations. The monotonic SST trends were then assessed for each summer ground using the *trend* package^29^.

### Abundance data

Narwhals have strong site fidelity to their coastal summer grounds and narwhal aggregations are defined based on their summer distribution^13,30^. Abundance estimates that were obtained in previous studies (from 11 of the 17 summer grounds, Table 1) where more than one abundance was available were used in the analysis. The methods used for obtaining the abundance estimates are described in Heide-Jørgensen^16^ and Doniol-Valcroze^17^. The estimates were used to relate whales’ abundance and density (whales/km^2^) to SST, and assess the effect of temperature on the abundance and densities of narwhals. SST data in August (month of the abundance surveys) were extracted at each summer ground for each sampled year. The relationships between abundance and SST and whale density and SST were then investigated using a linear non-parametric Theil-Sen regression model from the *mblm* package^31^. To assess the relation between SST change over time and the abundance of narwhals, the SST slope derived from each summer ground over the entire period (1993 to 2018) was related to the estimates of narwhal abundance and density separately.

**Table 1:** Abundance estimates for the 11 localities. SG refers to the summer ground’s abbreviation, CV to coefficient of variation and CR to the coefficient of regression of the SST trend over time extracted for each stock.

Stock	SG	Sector	Year	Area	Estimate	CV	Density	CR	SST	References
				(km^2^)	(whales)		(whales/km^2^)		(°C)	
Peel sound	PS	CAA	1996	14,735	5240	0.6	0.36	0.022	− 0.89	^55^
Peel sound	PS	CAA	2013	5454	17,003	0.21	3.12	0.039	0.39	^17^
Barrow strait	BS	CAA	1996	27,405	5898	0.75	0.22	0.022	− 1.05	^55^
Barrow strait	BS	CAA	2004	21,599	2925	0.46	0.14	0.022	− 0.69	^56^
Prince regent inlet	PRI	CAA	1996	29,296	34,159	0.35	1.17	0.036	0.33	^55^
Prince regent inlet	PRI	CAA	2002	30,628	20,871	0.71	0.68	0.036	− 0.62	^56^
Prince regent inlet	PRI	CAA	2013	29,178	10,668	0.63	0.37	0.036	1.29	^17^
Gulf of Bothia	GB	CAA	2002	56,567	6770	0.3	0.12	0.033	− 0.26	^56^
Gulf of Bothia	GB	CAA	2013	63,178	21,574	0.28	0.34	0.033	0.74	^17^
Admiralty inlet	AI	CAA	2003	9464	5362	0.5	0.57	0.011	2.22	^17^
Admiralty inlet	AI	CAA	2010	8159	18,049	0.23	2.21	0.011	2.24	^57^
Admiralty inlet	AI	CAA	2013	9419	35,043	0.42	3.72	0.011	2.59	^17^
Eclipse sound	ES	CAA	2004	6690	20,225	0.36	3.02	0.12	− 0.43	^56^
Eclipse sound	ES	CAA	2013	8459	10,489	0.24	1.24	0.12	2.13	^17^
East Baffin Island	EB	CAA	2003	11,204	10,073	0.31	0.9	0.13	0.49	^56^
East Baffin Island	EB	CAA	2013	53,510	17,555	0.35	0.33	0.13	4	^17^
Melville Bay	MB	Northwest	2012	14,821	2983	0.31	0.2	0.2	5.4	^58^
Melville Bay	MB	Northwest	2014	14,821	3091	0.5	0.21	0.2	4.66	^58^
Tasiilaq	TAS	Southeast	2008	2966	206	0.55	0.07	0.071	6.35	^59^
Tasiilaq	TAS	Southeast	2016	5401	0	0	0	0.071	9.49	^59^
Kangerlussuaq	KAN	Southeast	2008	672	613	0.71	0.31	0.11	5.34	^59^
Kangerlussuaq	KAN	Southeast	2016	667	269	0.37	0.06	0.11	8.33	^59^
Scoresby sound	SCS	Mideast	2008	11,144	1945	0.57	0.17	0.13	3.1	^59^
Scoresby sound	SCS	Mideast	2016	9828	433	0.49	0.04	0.13	6.04	^59^
Scoresby sound	SCS	Mideast	2017	7927	246	0.43	0.03	0.13	5.52	^59^

## Results

### Temperature affinities at the narwhals’ tracks

**Figure 1:**
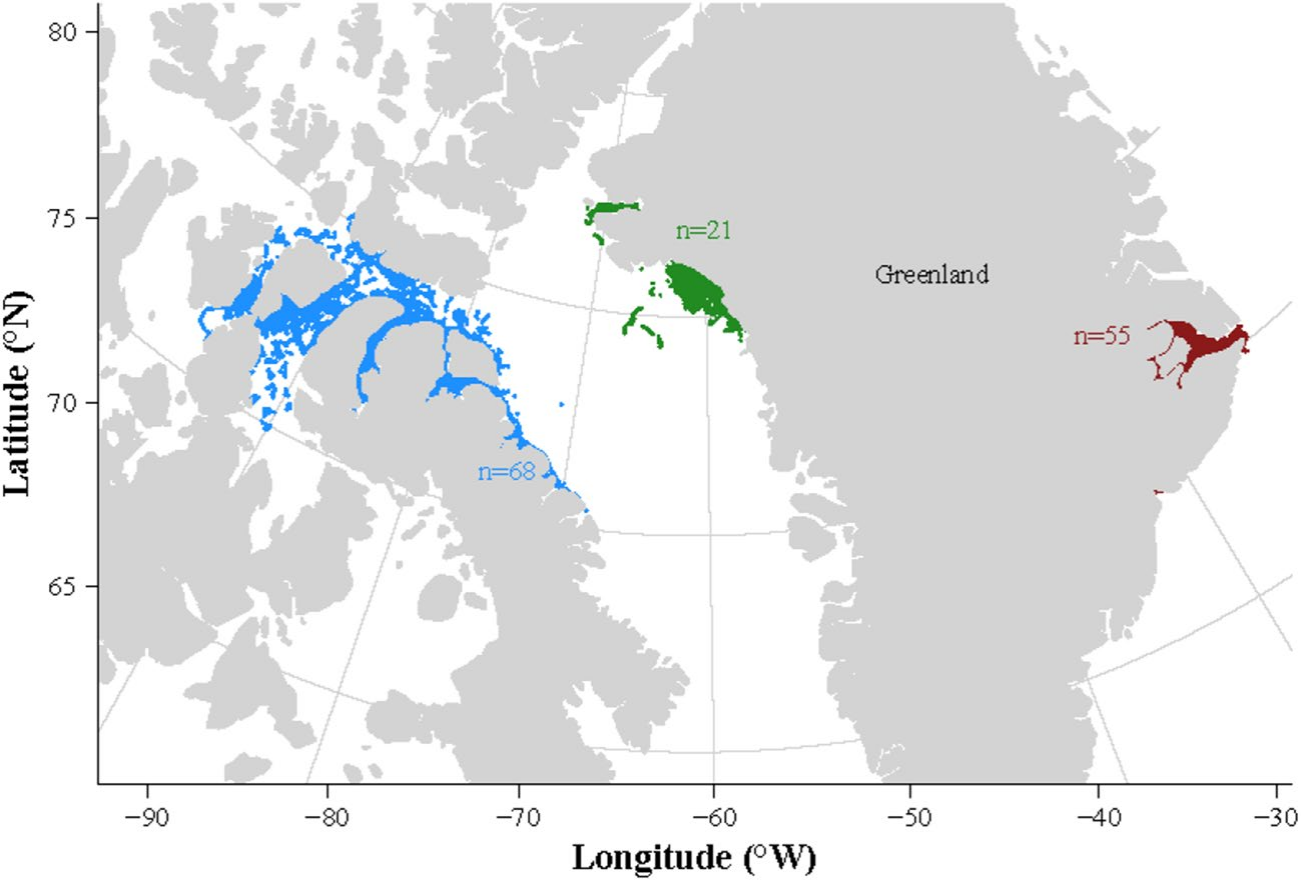
Summer distribution of narwhals’ tracks in the three tagging areas: the Canadian Arctic Archipelago, blue dots), West Greenland (green dots) and East Greenland (red dots). The number of tracked narwhals is indicated in each colour.

From 1993 to 2019, a total of 144 narwhals were satellite-tracked in summer in Canadian Arctic Archipelago (n = 68), West Greenland (n = 21) and East Greenland (n = 55)—see Fig. 1 and Table SI1.

In summer, narwhals tracked from populations in West Greenland and in the CAA dispersed widely across Baffin Bay, northern Baffin Island and within the fjords of the CAA. The narwhals that were tracked from populations in East Greenland remained close to or just south of the Scoresby Sound summer ground (Mideast of Greenland).

**Figure 2:**
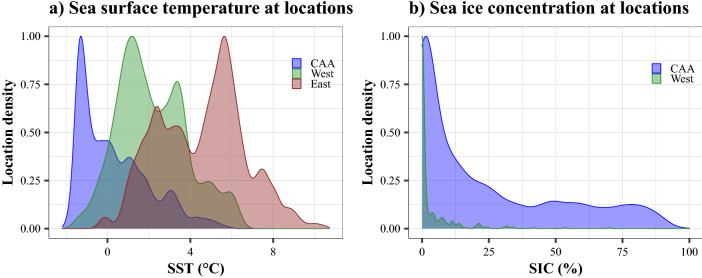
Histograms of (**a**) sea surface temperature and (**b**) sea ice concentration extracted at each narwhal location for CAA (in blue), West (in green) and East (in red) in summer.

The habitat encountered by the narwhals tracked from populations in the CAA, West and East Greenland differed in summer. Narwhals from West Greenland and CAA experienced significantly cooler sea surface temperatures during summer (ranging between − 2 and 4 °C, Kruskal–Wallis test, *p* < 0.001, Fig. 2a) associated with significantly higher sea-ice concentrations (up to > 99%, Fig. 2b, Kruskal–Wallis test, *p* < 0.001). By comparison, narwhals in East Greenland experienced significantly warmer SSTs (range: 0 to 10 °C, Kruskal–Wallis test, *p* < 0.001) and no sea-ice concentrations in summer (Fig. 2b).

### Temperature trends at the summer grounds

**Figure 3:**
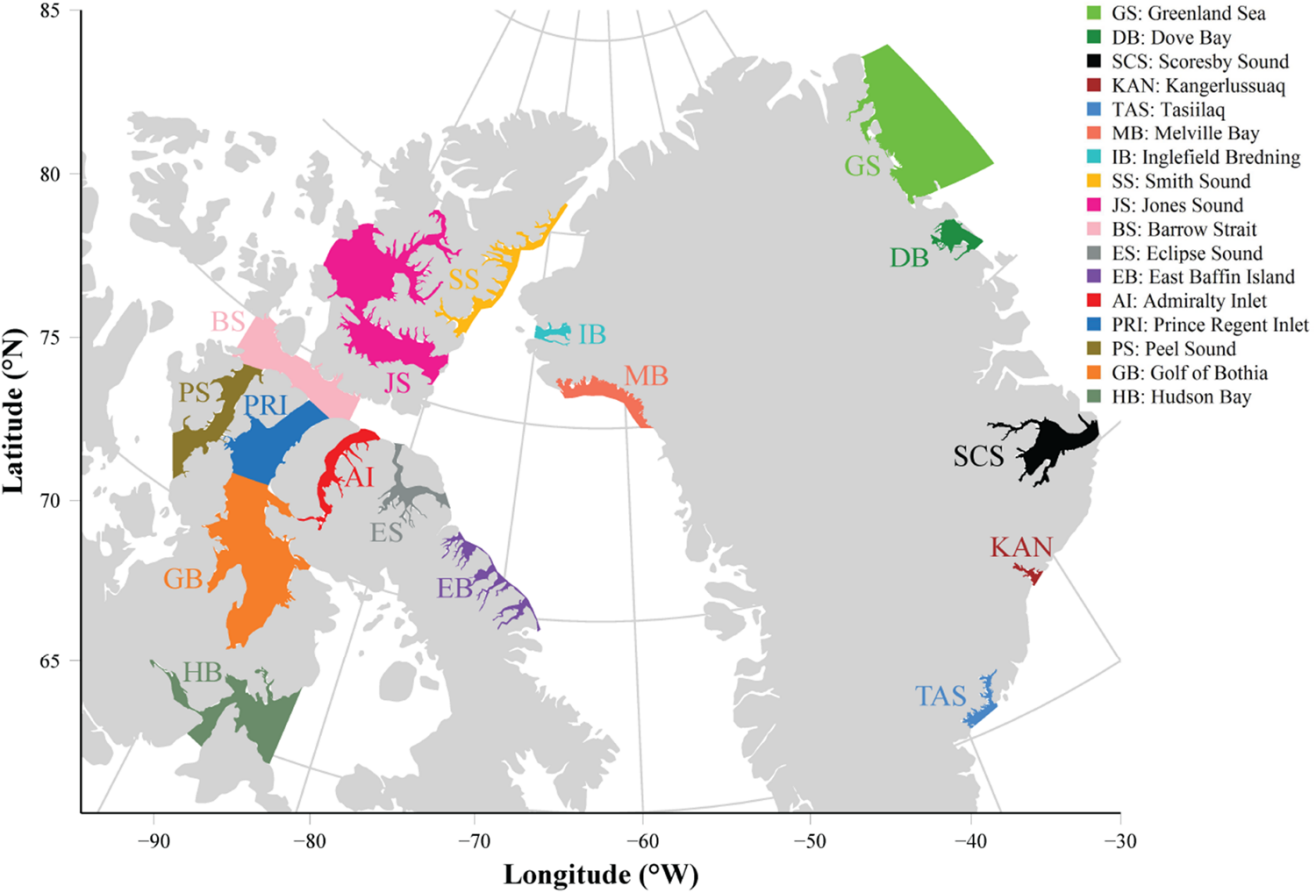
Map of 17 narwhal summer grounds in Canada and Greenland. The extent of the summer grounds reflects the summer distribution of whales as delimited by abundance surveys. Summer ground areas were used for estimating the annual trends in SST.

A total of 17 summer grounds are used by narwhal populations in Canada and Greenland (Fig. 3), with eight summer grounds (HB, GB, PS, PRI, BS, AI, ES and EB) in the central CAA, two in the northern part of the CAA (JS and SS), and two others in Northwest Greenland (IB and MB). In East Greenland there are two summer grounds (GS and DB) in the Northeast, one in the Mideast (SCS), and two in the Southeast Greenland (KAN and TAS).

**Figure 4:**
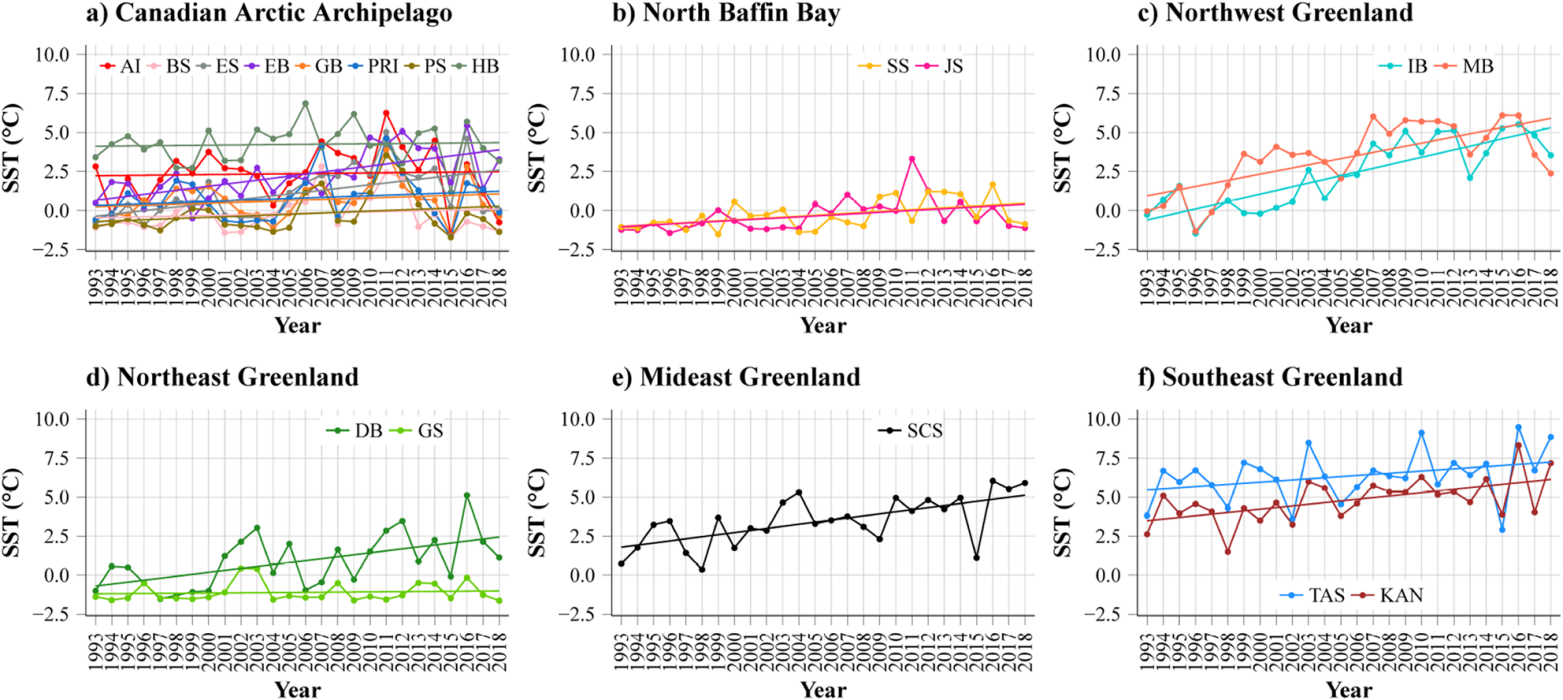
Trends in August SST values (means) between 1993 and 2018 for the summer grounds located in (**a**) the Canadian Arctic Archipelago (CAA), (**b**) North of Baffin Bay, (**c**) Northwest Greenland, (**d**) Northeast Greenland, (**e**) Mideast Greenland and (**f**) Southeast Greenland.

The SST trends on the summer grounds differed according to the areas (Fig. 4) and are presented in Table 2. In the central and northern parts of the CAA and Baffin Bay, SST was relatively stable across years. The Mann–Kendall tests confirmed this monotonic trend in the CAA, as it was non-significant for all localities except EB (Mann–Kendall test: *p* < 0.005) and ES (Mann–Kendall test: *p* < 0.005, Fig. 4a). The trend was significant in the two North Baffin Bay localities (SS, JS, Mann–Kendall test, *p* < 0.05, Fig. 4b). In contrast, SST increased over time in all localities in Northwest Greenland (IB, MB, Mann–Kendall test: *p* < 0.001, Fig. 4c), Mideast (SCS, Mann–Kendall test: *p* < 0.001, Fig. 4e) and Southeast Greenland (KAN, TAS, Mann–Kendall test for KAN and TAS: *p* < 0.001 and *p* = 0.1, respectively, Fig. 4f). Finally, in Northeast Greenland, the SST increased for DB (Mann–Kendall test: *p* < 0.005) whereas it was steady for GS (Mann–Kendall test: *p* = 0.48)—see Fig. 4d.

To test this hypothesis, we used a large dataset comprised of 144 satellite-tracked narwhals (Table SI1), sea surface temperatures (SST) data spanning 25 years (1993–2018) and population abundance estimates from 17 localities. This study aimed to (1) assess the thermal preference of this cold-adapted species, (2) investigate the temperature trends at its summer foraging grounds, and to finally (3) assess the relationship between sea temperatures and abundance of narwhals.

**Table 2:** Summary of the SST trends for each summer foraging ground (SG) with the associated *p* values.

Sector	SG	Trend	*p* value
CAA	AI	0.011x + 2.2	0.822
CAA	BS	0.022x − 0.49	0.493
CAA	EB	0.13x + 0.52	< 0.001
CAA	ES	0.12x − 0.48	< 0.005
CAA	GB	0.033x + 0.21	0.297
CAA	HB	0.0092x + 4.1	0.778
CAA	PRI	0.036x + 0.29	0.357
CAA	PS	0.039x − 0.75	0.246
North Baffin	JS	0.058x − 1.1	0.034
North Baffin	SS	0.06x − 1.1	0.011
Northwest	IB	0.24x − 0.84	5.232
Northwest	MB	0.2x + 0.74	< 0.001
Northeast	GS	0.0074x − 1.2	0.651
Northeast	DB	0.13x − 0.83	< 0.005
Mideast	SCS	0.13x + 1.7	< 0.001
Southeast	KAN	0.11x + 3.4	< 0.005
Southeast	TAS	0.071x + 5.4	0.092

### Relationship between SST and narwhal abundance

**Figure 5:**
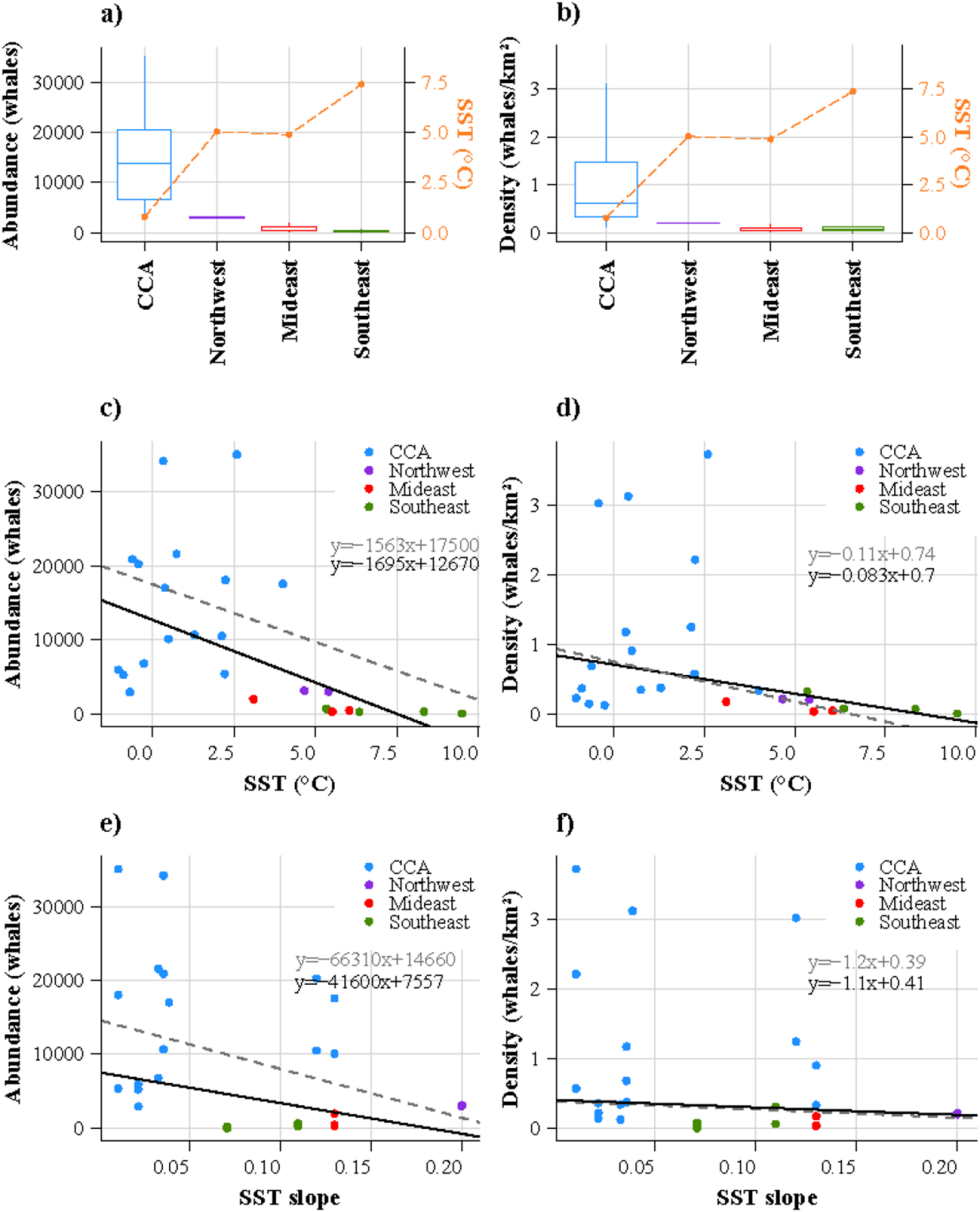
Boxplots of (**a**) narwhal abundance and (**b**) narwhal density for the four sectors where abundance data were available. The horizontal lines in the box plots refer to the medians, and the dotted orange lines in (**a**) and (**b**) to the mean SST for each sector. Scatterplots showing the relationship between narwhal abundance and (**c**) SST and (**e**) SST slope, and narwhal density and (**d**) SST and (**f**) SST slope. The Theil-Sen regression lines are shown in black and grey with the corresponding equation. The black lines refer to the Theil-Sen regressions including all estimates for each sector, and the grey dotted lines to the latest estimate for each locality. Each dot represents an abundance estimate from the 11 summer grounds that contained more than one estimate.

To increase the robustness of the results, the summer grounds containing less than two abundance estimates were not included in the analysis (HB, IB, SS, JS, DB and GS)—See Table 1. In the CAA, the SST was much lower compared to all other localities (mean ± SE: 0.7 ± 0.3 °C vs. 6.0 ± 0.6 °C, Fig. 5a). Whale abundance was also higher in the CAA (mean ± SE: 15,119 ± 2450 whales), and minimal in the Mideast (874 ± 537 whales) and Southeast Greenland (272 ± 127 whales) summer grounds (Fig. 5a). Similarly, narwhal density was highest in the CAA (1.15 ± 0.29 whales km^−2^), and lowest in the Mideast Greenland (0.08 ± 0.04 whales km^−2^, Fig. 5b).

Whale abundance was inversely related to SST (Fig. 5c). The whale density also decreased with increasing sea temperatures (Fig. 5d). For both relationships, the Theil-Sen regression model was significant (*p* < 0.001 for abundance and *p* < 0.05 for density). Similarly, Theil-Sen regressions were significant when considering only the latest estimate for each locality (Fig. 5c,d, grey dotted lines). When looking at the SST trend, the narwhals’ abundance and density decreased with SST slope evaluated over the entire period (1993–2018) (Fig. 5e,f).

## Discussion

### A narrow thermal preference

We have demonstrated that narwhal densities are highest for a narrow range of sea temperatures. These results are in agreement with a previous study conducted on bowhead whales (*Balaena mysticetus*) in the same region^32^, and suggest a limited thermal preference of Arctic cetaceans. Despite the limited thermal range on both sides of Greenland, the temperature exposure was four degrees higher in East Greenland compared to West Greenland and the Canadian Arctic Archipelago (CAA). Increasing water temperatures and sea-ice loss are expected to strongly modify the seasonality, distribution, and concentration of prey targeted by apex predators such as the narwhal and the bowhead whale, thus, potentially threatening the survival of these Arctic cetaceans^33^.

Narwhals feed on polar cod (*Boreogadus saida*), Arctic cod (*Arctogadus glacialis*) and squid (*Gonatus fabricii*) in all areas, and in Baffin Bay they also target Greenland halibut (*Reinhardtius hippoglossoides*)^34^. A recent study in the Chukchi Sea showed that the abundance of the small-size cod (one of the narwhal’s main prey) peaked between 4 and 5 °C^35^, and cold temperatures (< 2 °C) are known to favour the growth of the juvenile Arctic cod^36^. On the wintering grounds in Baffin Bay, narwhals are assumed to be feeding on Greenland halibut that are most abundant at cool bottom temperatures of 0.5–1.0 °C^37^. Over the long-term, increasing water temperature might therefore reduce the prey biomass available for narwhals, forcing this species to shift habitat or prey.

A question also remains regarding the direct consequence of oceanic warming on the physiology of this cold-adapted species by over-heating. With a thermal conductivity of water 25 times that of air, heat loss in marine mammals is much faster than in air of the same temperature^38^. Whales have a thick subcutaneous fat deposit (blubber) that prevents excessive heat loss and functions as thermal insulation and energy reserve^39^. Cetaceans dispose excess heat from poorly insulated peripheral areas, such as fins and tail flukes, that are utilized as thermal windows through which heat can be exchanged via conduction and convection^40^. Similar to bowhead whales, narwhals maintain thick blubber layer and lack a dorsal fin to serve as a thermal window for dissipating excess heat. As oceanic temperatures rise, these extreme adaptations that enable polar living may serve as a liability for narwhals, especially as the ice cover becomes more unpredictable^41^, and preferred prey move to new areas or depths. Their limited physiological flexibility also prevents them from adjusting their swimming and diving behaviour to climate change via shifts in habitats and prey selections^41^.

Studies of thermal habitat preference in narwhals have shown an extreme selection of a very narrow temperature range where the whales avoid water masses with temperatures above 2 °C^21^. The blubber insulation evidently protects the whales from hypothermia but the thermal niche also suggests that excess heat production during elevated locomotion activity can be a problem for the whales. Unlike bowhead whales, narwhals show a limited behavioural plasticity in their movement patterns^22,23,30^. Bursts of high levels of exercise and consequent heat production in connection with normal foraging behaviour or due to anthropogenic disturbance exacerbate the problem of temperature regulation. Together, thick blubber insulation, the lack of a dorsal fin coupled with a warming environment may limit the ability of Arctic cetaceans to remove excess heat and prevent hyperthermia. The strong site fidelity to their summer grounds might also limit their ability to adapt to a changing environment. Narwhals from East Greenland are in a particularly challenging situation due to the elevated temperature optimum compared to West Greenland and the CAA. Based on this, further studies are needed concerning the thermal physiology of Arctic cetaceans in general.

### Consequences of climate change on whale abundance

A sharp increase in sea temperature was observed in the Northwest, Mideast and Southeast of Greenland, whereas no change occurred in the CAA, which is in agreement with the predictions of thermal suitable habitats. Unlike the warm and saline Atlantic waters that are responsible for the rising temperatures in Northwest Greenland^18^, the cold CAA waters originate from the polar basin, ice melt and river discharges^42^, which explains the stable temperatures. Similarly to the Northwest, Mideast Greenland was characterized by a sharp temperature increase over time, as the result of warm Atlantic waters flowing northward between Iceland and East Greenland^20^. In addition, the southward transport of multiyear sea ice from Fram Strait along East Greenland has been severely reduced over the three past decades^43^.

Considering the extreme dependency of narwhals on cold water habitats, it is perhaps not surprising that the largest narwhal abundances are found in areas that meet these specifications, and that localities where increasing sea surface temperatures have been detected, host the smallest populations. Recent survey efforts have documented surprisingly large abundances of narwhals in areas that have not been surveyed before, but from where little or no historical evidence of narwhal concentrations is available. These areas include Dove Bay, Northeast Greenland, Smith Sound and Jones Sound, and although no systematic efforts to document narwhal populations in these areas have been conducted, the new abundance estimates indicate a possible shift in distribution. In Dove Bay, for instance, a 3-year expedition from 1906 to 1908 failed to detect any narwhals, whereas a survey conducted in 2017 estimated approximately 2000 narwhals in the bay^44^. Similarly, new abundance estimates of about 16,000 and 13,000 narwhals in the cold regions of Smith Sound and Jones Sound have not been documented before and these estimates are extremely high compared to past information from these areas^45–47^.

Trends in narwhal populations due to habitat changes are difficult to assess as some of the populations are also hunted, which affects population trends to varying degrees^48,49^. Trajectories of trends for narwhal populations in Mideast and Southeast Greenland show with high probability that these populations have never been large compared to populations in CAA and Northwest Greenland. Due to their low present abundance they are particularly vulnerable to overexploitation^13^. Southeast Greenland is at the same time the area with the most pronounced increases in SST. In comparison with the larger narwhal populations in the CAA and Northwest Greenland. The small size of the populations and increasing SST emphasizes the need for restrictive management of the exploitation of the populations in Mideast and Southeast Greenland. Independent of the history of exploitation, the magnitude of the differences in population sizes reflects the actual importance and habitat suitability of the localities. Of particular significance is the narwhal population in Southeast Greenland that is affected by a regime shift in oceanography with warming temperatures in coastal areas and a reduced transport of polar pack-ice along the East Greenland coast. Although excessive hunting occurs in Southeast Greenland, the decline or disappearance of narwhals from previously used summer grounds and the shift of hunting effort to more northern and presumably colder areas indicates that Southeast Greenland may no longer be a suitable habitat for narwhals^50^.

## Conclusion

By combining telemetry data to abundance estimates and sea surface temperatures we were able to assess (1) the thermal exposure, (2) the SST trends at the summer foraging grounds, and (3) the relationship between SST and abundance of a cold-adapted species, the narwhal. Despite the observation of widespread increase in temperature over the Arctic, the SST time series (from 1993 to 2018) indicate different trends across the summer grounds, with a noticeable correlation between increasing sea temperatures and the whales’ abundance. These results support the hypothesis that warming ocean waters will restrict the habitat range of this Arctic cetacean, further suggesting that narwhals from Mideast and Southeast Greenland may be under pressure to abandon their traditional habitats due to ocean warming, and consequently either migrate further North, or locally go extinct. In addition to indirect effects on their distribution, rising temperatures may affect the whales through physiological stress. To survive in cold environments, polar marine species have developed morphological adaptations to provide thermal insulation through dense plumage, thick fur^51^ or a blubber layer^52^. Cetaceans endemic to the Arctic also lack a dorsal fin, which in boreal species serves to dissipate heat^53^. These morphological specialisations allow travel in ice-covered areas, but may represent a morphological handicap for transferring excess body heat compared to sub-Arctic cetacean species^54^ such as the harbour porpoise and humpback whale. Warmer temperatures could therefore represent a potential thermal challenge for Arctic cetaceans with no dorsal fin such as narwhals and bowhead whales, and warrants further investigation.

## Supplementary information

Supplementary Information
