# Supplementary material for: The impact of rising sea temperatures on an Arctic top predator, the narwhal
Source: Sci Rep. 2020 Oct 29;10:18678. doi: 10.1038/s41598-020-75658-6 (PMC7596713; doi:10.1038/s41598-020-75658-6)
Supplement: Supplementary file 1 — Supplementary Information [file 41598_2020_75658_MOESM1_ESM.docx]

**SUPPORTING INFORMATION**

**The impact of rising sea temperatures on an Arctic top predator, the narwhal**

P. Chambault^1^, O.M. Tervo^1^, E. Garde^1^, R.G. Hansen^1^, S.B. Blackwell^2^, T.M. Williams^3^, R. Dietz^4^, C.M. Albertsen^5^, K.L. Laidre^6^, N.H. Nielsen ^7^, P. Richard^8^, M.H.S. Sinding^1,9^, H.C. Schmidt^1^, M.P. Heide-Jørgensen^1^

**Table SI 1.** List of satellite tracked narwhals from 1993-2019 tagged in Canada and Greenland. 1 stands for the whale’s ID and Nloc to the number of locations recorded in summer.

| **PTT** | **Sector** | | **Start date** | **End date** | **Nloc** | **Distance** | **Duration** | **Speed** |
| --- | --- | --- | --- | --- | --- | --- | --- | --- |
|  |  |  |  |  |  | **(km)** | **(days)** | **(km/h)** |
| 1993MM1 | West | 02/09/1993 | | 30/09/1993 | 238 | 2526 | 29 | 4.7±2.1 |
| 1993MM3 | West | 03/09/1993 | | 30/09/1993 | 19 | 300 | 27 | 3.9±2.9 |
| 1993MM5 | West | 23/08/1993 | | 29/09/1993 | 126 | 206 | 37 | 0.3±0.2 |
| 1994MM1 | West | 27/08/1994 | | 30/09/1994 | 483 | 3026 | 34 | 3.9±1.7 |
| 1994MM2 | West | 25/08/1994 | | 19/09/1994 | 214 | 1832 | 25 | 3.7±2.2 |
| 1994MM3 | West | 27/08/1994 | | 12/09/1994 | 57 | 1158 | 16 | 4.5±2.4 |
| 1994MM4 | West | 25/08/1994 | | 30/09/1994 | 376 | 1672 | 36 | 2.1±1.3 |
| 1997MM1 | CAA | 23/08/1997 | | 25/08/1997 | 11 | 145 | 2 | 3.8±2.2 |
| 1997MM2 | CAA | 22/08/1997 | | 05/09/1997 | 179 | 626 | 14 | 2.2±1.5 |
| 1997MM3 | CAA | 24/08/1997 | | 30/09/1997 | 562 | 2428 | 37 | 3.1±1.9 |
| 1997MM4 | CAA | 10/08/1997 | | 10/09/1997 | 102 | 2551 | 31 | 4.1±2.8 |
| 1997MM5 | CAA | 22/08/1997 | | 09/09/1997 | 26 | 237 | 18 | 2.4±2.8 |
| 1998MM1 | CAA | 14/08/1998 | | 26/08/1998 | 8 | 42 | 12 | 0.2±0.2 |
| 1998MM2 | CAA | 26/08/1998 | | 30/09/1998 | 35 | 965 | 35 | 1.1±1.1 |
| 1998MM3 | CAA | 21/08/1998 | | 30/09/1998 | 41 | 881 | 40 | 0.9±0.8 |
| 1998MM4 | CAA | 19/08/1998 | | 20/09/1998 | 69 | 723 | 32 | 2.6±2.8 |
| 1998MM5 | CAA | 26/08/1998 | | 30/09/1998 | 36 | 576 | 35 | 0.6±0.5 |
| 1999MM1 | CAA | 12/08/1999 | | 30/09/1999 | 845 | 4257 | 49 | 4.4±2.2 |
| 1999MM2 | CAA | 13/08/1999 | | 30/09/1999 | 1236 | 2482 | 48 | 2.5±1.8 |
| 1999MM3 | CAA | 15/08/1999 | | 30/09/1999 | 1272 | 3681 | 46 | 3.8±2.2 |
| 1999MM4 | CAA | 15/08/1999 | | 30/09/1999 | 1421 | 3128 | 46 | 3.3±2.1 |
| 1999MM5 | CAA | 21/08/1999 | | 27/08/1999 | 149 | 113 | 5 | 1±0.62 |
| 1999MM6 | CAA | 21/08/1999 | | 30/09/1999 | 1145 | 2643 | 40 | 3.3±2 |
| 1999MM7 | CAA | 21/08/1999 | | 30/09/1999 | 1266 | 3374 | 40 | 3.9±2.3 |
| 2000MM1 | CAA | 14/08/2000 | | 30/09/2000 | 1714 | 2230 | 47 | 2±1.3 |
| 2000MM10 | CAA | 17/08/2000 | | 25/08/2000 | 146 | 286 | 8 | 1.9±1.3 |
| 2000MM2 | CAA | 14/08/2000 | | 30/09/2000 | 1862 | 3646 | 47 | 3.2±1.6 |
| 2000MM3 | CAA | 25/08/2000 | | 23/09/2001 | 258 | 1414 | 394 | 2.2±1.2 |
| 2000MM4 | CAA | 25/08/2000 | | 09/09/2001 | 188 | 1150 | 381 | 2.5±1.6 |
| 2000MM5 | CAA | 17/08/2000 | | 30/09/2000 | 358 | 1835 | 44 | 1.9±1.1 |
| 2000MM6 | CAA | 17/08/2000 | | 30/09/2000 | 432 | 2601 | 44 | 2.8±1.6 |
| 2000MM7 | CAA | 13/08/2000 | | 30/09/2000 | 547 | 1978 | 48 | 2±1.4 |
| 2000MM8 | CAA | 17/08/2000 | | 30/09/2000 | 621 | 2195 | 43 | 2.5±1.7 |
| 2000MM9 | CAA | 19/08/2000 | | 01/09/2000 | 289 | 680 | 14 | 2.2±1.2 |
| 2001MM1 | CAA | 15/08/2001 | | 25/09/2001 | 71 | 348 | 41 | 2.2±1.5 |
| 2001MM2 | CAA | 15/08/2001 | | 25/08/2001 | 26 | 199 | 10 | 2.6±1.9 |
| 2001MM3 | CAA | 15/08/2001 | | 25/09/2001 | 216 | 420 | 42 | 1.9±1.2 |
| 2001MM4 | CAA | 10/08/2001 | | 26/09/2001 | 206 | 521 | 46 | 2.2±1.1 |
| 2001MM5 | CAA | 15/08/2001 | | 25/09/2001 | 206 | 758 | 42 | 2.9±1.5 |
| 2001MM6 | CAA | 15/08/2001 | | 25/08/2001 | 76 | 252 | 11 | 1.7±1.1 |
| 2001MM7 | CAA | 10/08/2001 | | 25/09/2001 | 204 | 1166 | 46 | 2.8±1.5 |
| 2003MM1 | CAA | 17/08/2003 | | 30/09/2003 | 543 | 2316 | 44 | 3.8±2.2 |
| 2003MM10 | CAA | 22/08/2003 | | 28/09/2003 | 111 | 761 | 37 | 1.6±1.2 |
| 2003MM11 | CAA | 21/08/2003 | | 30/09/2003 | 490 | 732 | 40 | 1.3±0.9 |
| 2003MM12 | CAA | 17/08/2003 | | 28/09/2003 | 146 | 1343 | 42 | 3.5±2.1 |
| 2003MM13 | CAA | 21/08/2003 | | 15/09/2003 | 228 | 1016 | 24 | 3.5±2 |
| 2003MM2 | CAA | 19/08/2003 | | 28/09/2003 | 130 | 954 | 40 | 3.2±1.8 |
| 2003MM3 | CAA | 16/08/2003 | | 30/09/2003 | 598 | 2389 | 45 | 3.5±1.9 |
| 2003MM4 | CAA | 16/08/2003 | | 30/09/2003 | 535 | 1701 | 45 | 3±1.7 |
| 2003MM5 | CAA | 18/08/2003 | | 30/09/2003 | 692 | 1686 | 43 | 1.6±1 |
| 2003MM6 | CAA | 14/08/2003 | | 30/09/2003 | 822 | 2728 | 47 | 3.3±1.6 |
| 2003MM7 | CAA | 17/08/2003 | | 30/09/2003 | 506 | 2235 | 44 | 3.4±2.1 |
| 2003MM8 | CAA | 17/08/2003 | | 14/09/2003 | 700 | 1623 | 27 | 3.6±2.2 |
| 2003MM9 | CAA | 22/08/2003 | | 28/09/2003 | 110 | 786 | 37 | 3.3±2.1 |
| 2004MM1 | West | 06/09/2004 | | 28/09/2004 | 236 | 767 | 22 | 2.6±1.7 |
| 2004MM10 | CAA | 25/08/2004 | | 30/09/2004 | 65 | 806 | 36 | 3.5±2.3 |
| 2004MM11 | CAA | 24/08/2004 | | 26/09/2004 | 30 | 248 | 34 | 2.2±2.1 |
| 2004MM2 | West | 07/09/2004 | | 22/09/2004 | 33 | 456 | 15 | 2.7±2.5 |
| 2004MM3 | West | 06/09/2004 | | 26/09/2004 | 36 | 229 | 20 | 1.6±2.1 |
| 2004MM4 | CAA | 09/08/2004 | | 16/09/2004 | 193 | 539 | 38 | 1.3±1.1 |
| 2004MM5 | CAA | 09/08/2004 | | 30/09/2004 | 291 | 1288 | 52 | 2.7±1.9 |
| 2004MM6 | CAA | 13/08/2004 | | 30/09/2004 | 285 | 1151 | 48 | 2.2±1.6 |
| 2004MM7 | CAA | 15/08/2004 | | 30/09/2004 | 248 | 1067 | 46 | 2.5±1.7 |
| 2004MM9 | CAA | 25/08/2004 | | 30/09/2004 | 43 | 564 | 36 | 1.4±0.8 |
| 2005MM1 | West | 02/09/2005 | | 18/09/2005 | 177 | 402 | 16 | 2.6±1.3 |
| 2005MM10 | CAA | 18/08/2005 | | 30/09/2005 | 531 | 1745 | 43 | 2.3±1.7 |
| 2005MM11 | CAA | 18/08/2005 | | 13/09/2005 | 154 | 580 | 26 | 2.5±1.8 |
| 2005MM12 | CAA | 18/08/2005 | | 28/09/2005 | 289 | 782 | 41 | 2.5±1.9 |
| 2005MM13 | CAA | 21/08/2005 | | 28/09/2005 | 115 | 1522 | 39 | 3.3±2.1 |
| 2005MM14 | CAA | 21/08/2005 | | 31/08/2005 | 17 | 89 | 10 | 0.52±0.68 |
| 2005MM2 | CAA | 14/08/2005 | | 30/09/2005 | 266 | 845 | 47 | 2.8±2 |
| 2005MM3 | CAA | 14/08/2005 | | 30/09/2005 | 276 | 1128 | 47 | 2.9±1.6 |
| 2005MM4 | CAA | 17/08/2005 | | 02/09/2005 | 123 | 227 | 16 | 1.7±1.2 |
| 2005MM5 | CAA | 17/08/2005 | | 30/09/2005 | 249 | 939 | 44 | 3.1±1.7 |
| 2005MM6 | CAA | 18/08/2005 | | 30/09/2005 | 191 | 1170 | 44 | 4.3±2.8 |
| 2005MM7 | CAA | 18/08/2005 | | 30/09/2005 | 246 | 1311 | 44 | 4.3±2.2 |
| 2005MM8 | CAA | 18/08/2005 | | 30/09/2005 | 240 | 1215 | 44 | 3.5±2.2 |
| 2005MM9 | CAA | 21/08/2005 | | 30/09/2005 | 232 | 910 | 40 | 4.0±2.1 |
| 2006MM2 | West | 10/09/2006 | | 30/09/2006 | 129 | 589 | 21 | 2.8±1.9 |
| 2006MM3 | West | 10/09/2006 | | 30/09/2006 | 128 | 521 | 21 | 2.9±1.3 |
| 2006MM4 | West | 14/09/2006 | | 30/09/2006 | 135 | 425 | 17 | 2.6±1.5 |
| 2007MM10 | West | 26/08/2007 | | 30/09/2007 | 906 | 1254 | 35 | 1.5±1.3 |
| 2007MM3 | West | 07/08/2007 | | 10/08/2007 | 16 | 29 | 3 | 2.8±2.6 |
| 2007MM7 | West | 26/08/2007 | | 30/09/2007 | 592 | 1522 | 35 | 2.9±1.5 |
| 2007MM8 | West | 04/09/2007 | | 30/09/2007 | 395 | 1139 | 27 | 3.2±2.2 |
| 2007MM9 | West | 04/09/2007 | | 30/09/2007 | 221 | 644 | 26 | 3.8±1.9 |
| 2008MM10 | CAA | 02/07/2009 | | 24/07/2009 | 238 | 947 | 22 | 2.5±1.2 |
| 2008MM4 | West | 09/08/2008 | | 21/08/2008 | 70 | 107 | 12 | 1.3±1.6 |
| 2012MM2 | West | 25/08/2012 | | 20/09/2012 | 160 | 486 | 26 | 3.4±2.0 |
| deploy_10946 | East | 19/08/2011 | | 30/09/2011 | 676 | 2053 | 42 | 3.5±1.4 |
| deploy_20158 | East | 14/08/2015 | | 28/09/2015 | 1947 | 2022 | 45 | 3.6±1.8 |
| deploy_20162 | East | 13/08/2011 | | 30/09/2011 | 681 | 2166 | 49 | 3.2±1.6 |
| deploy_20685 | East | 17/08/2013 | | 30/09/2013 | 352 | 1317 | 44 | 4.8±2.6 |
| deploy_20696 | East | 11/08/2014 | | 30/09/2014 | 395 | 1952 | 50 | 4.5±2.5 |
| deploy_21791 | East | 23/08/2012 | | 30/09/2012 | 923 | 2460 | 38 | 3.9±2.1 |
| deploy_21792 | East | 19/08/2012 | | 30/09/2012 | 1161 | 1802 | 42 | 2.7±1.6 |
| deploy_22849 | East | 17/08/2012 | | 28/09/2012 | 1162 | 2848 | 42 | 3.2±1.9 |
| deploy_22850 | East | 19/08/2012 | | 30/09/2012 | 1928 | 3231 | 43 | 3.4±2.0 |
| deploy_22853 | East | 18/08/2012 | | 22/09/2012 | 1629 | 2617 | 35 | 3.1±1.8 |
| deploy_24638 | East | 16/08/2012 | | 21/09/2012 | 1381 | 1783 | 36 | 2.5±1.6 |
| deploy_27262 | East | 24/08/2016 | | 30/09/2016 | 884 | 2487 | 38 | 2.9±1.4 |
| deploy_37227 | East | 24/08/2016 | | 30/09/2016 | 602 | 1956 | 37 | 4.4±2.6 |
| deploy_3960 | East | 02/09/2010 | | 30/09/2010 | 377 | 1161 | 28 | 3.0±1.4 |
| 3962_2014 | East | 11/08/2014 | | 27/09/2014 | 244 | 1027 | 47 | 3.1±1.7 |
| 3962_2016 | East | 24/08/2016 | | 30/09/2016 | 2406 | 3291 | 37 | 4.0±1.6 |
| deploy_3963 | East | 22/08/2010 | | 30/09/2010 | 448 | 1608 | 39 | 3.6±1.6 |
| deploy_3964 | East | 02/09/2010 | | 30/09/2010 | 290 | 912 | 28 | 2.9±1.3 |
| deploy_3965 | East | 15/08/2015 | | 29/09/2015 | 642 | 1442 | 45 | 3.2±1.8 |
| deploy_50683 | East | 11/08/2014 | | 29/09/2014 | 329 | 1791 | 49 | 4.4±2.7 |
| deploy_6335 | East | 04/09/2010 | | 30/09/2010 | 352 | 1165 | 26 | 3.5±1.6 |
| 6335_2015 | East | 15/08/2015 | | 29/09/2015 | 500 | 1364 | 46 | 2.6±1.9 |
| deploy_6336 | East | 12/08/2011 | | 30/09/2011 | 697 | 2053 | 49 | 3.3±1.6 |
| deploy_7617 | East | 14/08/2014 | | 30/09/2014 | 906 | 3510 | 47 | 4.6±2.5 |
| deploy_7618 | East | 14/08/2015 | | 29/09/2016 | 919 | 4240 | 412 | 4.7±2.7 |
| deploy_7926 | East | 13/08/2011 | | 30/09/2011 | 640 | 2186 | 49 | 3.6±1.6 |
| 7926_2014 | East | 14/08/2014 | | 30/09/2014 | 1381 | 3096 | 47 | 3.7±2.0 |
| deploy_7934 | East | 14/08/2015 | | 28/09/2015 | 2531 | 2601 | 45 | 3.9±1.9 |
| deploy_93093 | East | 04/09/2010 | | 30/09/2010 | 268 | 945 | 26 | 3.1±1.8 |
| deploy_93094 | East | 04/09/2010 | | 30/09/2010 | 339 | 1042 | 26 | 3.4±1.7 |
| deploy_93095 | East | 17/08/2011 | | 30/09/2011 | 477 | 1811 | 45 | 3±1.3 |
| deploy_93096 | East | 08/08/2013 | | 02/09/2013 | 400 | 1219 | 25 | 4.8±2.3 |
| deploy_93097 | East | 17/08/2013 | | 30/09/2013 | 522 | 1713 | 44 | 4.7±2.6 |
| deploy_93098 | East | 13/08/2011 | | 30/09/2011 | 725 | 2120 | 48 | 3.5±1.5 |
| deploy_93099 | East | 14/08/2015 | | 29/09/2015 | 340 | 1619 | 46 | 4.7±2.6 |
| deploy_93101 | East | 19/08/2011 | | 30/09/2011 | 536 | 1950 | 42 | 3.6±1.7 |
| deploy_93102 | East | 17/08/2013 | | 30/09/2013 | 836 | 2088 | 44 | 4.4±2.2 |
| deploy_Mara | East | 11/08/2014 | | 30/09/2014 | 1142 | 3341 | 50 | 4.0±2.3 |
| deploy_Thora | East | 11/08/2014 | | 30/09/2014 | 1443 | 3722 | 50 | 4.2±2.1 |
| 22853 | East | 11/08/2017 | | 26/09/2017 | 1290 | 2238 | 46 | 4.0±1.7 |
| 168433 | East | 23/08/2018 | | 30/09/2018 | 4701 | 3583 | 39 | 4.6±1.8 |
| 168434 | East | 12/08/2017 | | 26/08/2017 | 277 | 299 | 14 | 5.3±1.8 |
| 168435 | East | 11/08/2017 | | 26/09/2017 | 3044 | 2633 | 46 | 4.6±2 |
| 168436 | East | 25/08/2018 | | 30/09/2018 | 5040 | 3825 | 37 | 4.7±2 |
| 168437 | East | 23/08/2018 | | 30/09/2018 | 4879 | 4034 | 38 | 4.7±1.9 |
| Hildur | East | 22/08/2017 | | 30/09/2017 | 170 | 2497 | 39 | 3.1±1.6 |
| 20165 | East | 11/08/2017 | | 26/09/2017 | 3023 | 2643 | 46 | 4.5±2.0 |
| 20158 | East | 23/08/2018 | | 30/09/2018 | 4848 | 3802 | 38 | 4.6±1.9 |
| 20160 | East | 24/08/2018 | | 30/09/2018 | 4408 | 3731 | 37 | 4.4±1.9 |
| 20162 | East | 12/08/2017 | | 26/09/2017 | 359 | 1359 | 46 | 5.5±2.4 |
| 37282 | East | 23/08/2017 | | 30/09/2017 | 172 | 2640 | 38 | 3.4±1.5 |
| 21791 | East | 23/08/2018 | | 03/09/2018 | 1869 | 1077 | 11 | 4.4±2.1 |
| 21793 | East | 26/08/2018 | | 30/09/2018 | 148 | 1920 | 35 | 2.8±1.5 |
| 22849 | East | 22/08/2017 | | 26/09/2017 | 502 | 1327 | 35 | 4.3±1.7 |
| 20696 | East | 25/08/2018 | | 30/09/2018 | 177 | 2114 | 36 | 3.0±1.8 |
|  |  |  | |  | *686+614* | *1601+874* | *43+17* | *3.7+2* |
